# Room-temperature nonequilibrium growth of controllable ZnO nanorod arrays

**DOI:** 10.1186/1556-276X-6-477

**Published:** 2011-07-27

**Authors:** Qian Li, Kui Cheng, Wenjian Weng, Chenlu Song, Piyi Du, Ge Shen, Gaorong Han

**Affiliations:** 1 Department of Materials Science & Engineering, State Key Laboratory of Silicon Materials, Zhejiang University, Hangzhou 310027, People's Republic of China

## Abstract

In this study, controllable ZnO nanorod arrays were successfully synthesized on Si substrate at room temperature (approx. 25°C). The formation of controllable ZnO nanorod arrays has been investigated using growth media with different concentrations and molar ratios of Zn(NO_3_)_2 _to NaOH. Under such a nonequilibrium growth condition, the density and dimension of ZnO nanorod arrays were successfully adjusted through controlling the supersaturation degree, i.e., volume of growth medium. It was found that the wettability and electrowetting behaviors of ZnO nanorod arrays could be tuned through variations of nanorods density and length. Moreover, its field emission property was also optimized by changing the nanorods density and dimension.

## Introduction

One-dimensional (1D) nanostructures, such as nanorods, nanowires, and nanotubes, have extensively been investigated in recent years for their excellent optoelectronic, electronic, mechanical, magnetic, photochemical properties, etc. [1-6]. The density, morphology, crystal size, and dimension of 1D nanostructure arrays are very important as they strongly determine the properties of the arrays, such as wettability, field emission property, photochemical property, etc. [7-9]. Müller et al. [10] have found that the contact angle increased as the density of Ge nanopyramids grown on Si substrate increased. Lau et al. [11] studied the wettability of poly-coated carbon nanotube array and demonstrated the contact angle increased when the height of nanotube increased. Among various nanomaterials, zinc oxide (ZnO) nanostructure has attracted much attention mainly because of its wide direct band gap (3.37 eV), large excitation binding energy (60 meV), optical transparency, electric conductivity, piezoelectricity, and so on [12]. Especially, many studies have been carried out on 1D ZnO nanorod because of its novel physical properties and potential applications in various nanostructure devices [13-19]. Numerous techniques to fabricate ZnO nanorod arrays on substrates have been reported, including metal-organic chemical vapor deposition [16], vapor transport process [17], solution chemical route [18-21], molecular beam epitaxy [22], pulsed laser deposition [23], electrochemical deposition [24], and thermal evaporation [25]. Among them, room temperature solution route is particularly attractive because of its low cost, facile synthesis, high efficiency, and various substrates choices. Several techniques have been reported for growing ZnO nanorods at room temperature. Electrochemical deposition technique is one among them [26], but this method is restricted to electrode area or the substrate property. In addition, wet chemical route to fabricate dense ZnO nanorods array on Zn foil [27] at room temperature has been reported, which is not applicable to various substrates.

The properties of ZnO nanorods are greatly influenced by the density, morphology and dimension of the arrays [28-31]. Therefore, room temperature preparation of ZnO nanorods arrays synthesized on different substrates with controllable morphologies and densities is quite required as it could favor and speed up the applications of 1D ZnO nanostructures. Meanwhile, the specialty of room temperature solution method is that the whole formation processes of ZnO nanostructures are out of equilibrium. The dynamic variations of supersaturation degree strongly influence the process of nonequilibrium growth in the solution. Although nanostructures could be synthesized by this nonequilibrium growth method, the nanostructure arrays with controlled morphology, density, and dimension are difficult to achieve.

In this study, the room temperature (approx. 25°C) solution growth of density- and dimension-controlled ZnO nanorod arrays in a nonequilibrium condition was studied. The formation of ZnO nanorod arrays was influenced by the concentration of growth medium and the molar ratio of Zn(NO_3_)_2 _to NaOH. The ZnO nanorod arrays with variable densities and dimensions were induced by tuning the volume of the growth medium, which resulted in a dynamic variations process of supersaturation degree. The wettability, electrowetting, and field emission properties of the ZnO nanorod arrays with different density and dimension were measured and discussed.

## Experimental

Clean Si (100) wafers (10 × 10 mm^2^) were used as substrates. For the preparation of ZnO seed-layer, 0.1-M zinc acetate dihydrate with ethanolamine in equimolar of Zn^2+ ^was dissolved into 10-mL ethanol solution and stirred continuously for 20 min to be precursor solution. 15-μL precursor solution aforesaid was spin-coated onto a clean Si substrate at 6000 rpm for 30 s. A seed-layer based on a Si substrate was formed after calcination at 500°C for 60 min. Then, an alkali growth medium containing Zn(NO_3_)_2 _and NaOH aqueous solution for growing ZnO nanorods was prepared following Zn(NO_3_)_2 _concentrations and molar ratios (*R*) of Zn(NO_3_)_2 _to NaOH tabulated in Table 1. Si substrates with seed-layers were vertically dipped in different volumes of growth medium solutions. After reaction at room temperature (approx. 25°C) for 2 h, the substrates were rinsed with ethanol and deionized water thoroughly, final products were obtained after drying in air, as shown in Table 1 (samples A-E_3_).

**Table 1:** Preparation conditions for different samples

Zinc concentration (M)	*R *(molar ratio of Zn^2+^/OH^-^)	Medium volume (mL)	Sample Name
0.25	1:8	2	A
0.25	1:8	10	B
0.25	1:8	20	C
0.25	1:4	20	D_1_
0.25	1:6	20	D_2_
0.25	1:10	20	D_3_
0.025	1:8	20	E_1_
0.05	1:8	20	E_2_
0.1	1:8	20	E_3_

Morphologies of the ZnO nanorods were observed in a field emission scanning electron microscope (Hitachi, S-4800). Phase and crystallinity of the nanorods were collected with an X-ray diffractometer (XRD, PANalytical, X'Pert PRO). The detailed structures of ZnO nanorods were investigated with a transmission electron microscope (TEM, JEOL, JEM-2010). The concentration of Zn^2+ ^was determined by atomic absorption spectroscopy (AAS, Hitachi, 180-80). The wettability and electrowetting behaviors were measured by water contact angle measurement (OCA 20, DATAPHYSICS) at room temperature. The electrowetting process relied on the modification of contact angle by the application of a voltage in the 0-60 V range between the doped Si substrate and the 3-μL droplet of deionized water. A thin Cu wire was used to achieve electrical contact with the droplet. The measurements of field emission were performed in a vacuum chamber with a pressure of about 2 × 10^-4 ^Pa at room temperature and the ZnO nanorods prepared on Si substrates were used as the cathode. The distance between the cathode and the anode was 600 μm and the measured field emission area was 0.48 cm^2^.

## Results and discussions

### Controlled growth of ZnO nanorod arrays

**Figure 1:**
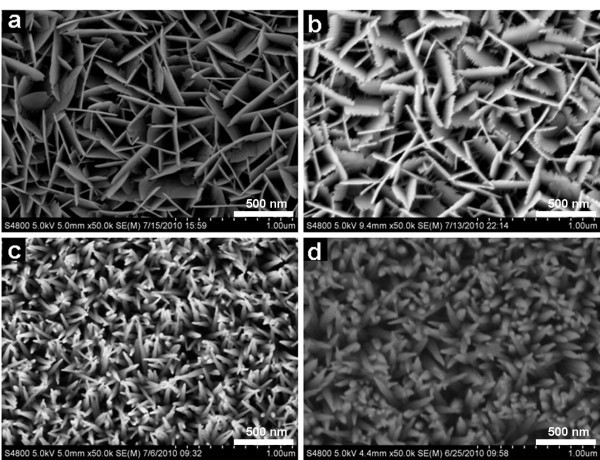
**SEM images of ZnO nanostructures grown by using different molar ratios of Zn^2+ ^to OH^-^**: **(a) **1:4; **(b) **1:6; **(c) **1:8; **(d) **1:10.

There were several parameters affecting the growth characteristics of ZnO nanorod arrays, such as molar ratio of Zn^2+ ^to OH^-^, zinc ion concentration, and volume of the growth medium (absolute quantity of zinc ion). Figure 1 shows the SEM pictures of samples D_1_, D_2_, C, and D_3_, which reveal the effects of different *R *ratios on nanorods morphology. When *R *was high (1:4 and 1:6), nanosheets formed (Figure 1a,b). It is noteworthy that small tips appear on the edge of nanosheets when *R *is 1:6. It is considered that these tips show the transformation of nanostructure from nanosheet to nanorod. When *R *decreased to 1:8, uniform ZnO nanorods with sharp tips formed (Figure 1c). Further decreasing *R *to 1:10 led to the formation of wider nanorods (Figure 1d).

**Figure 2:**
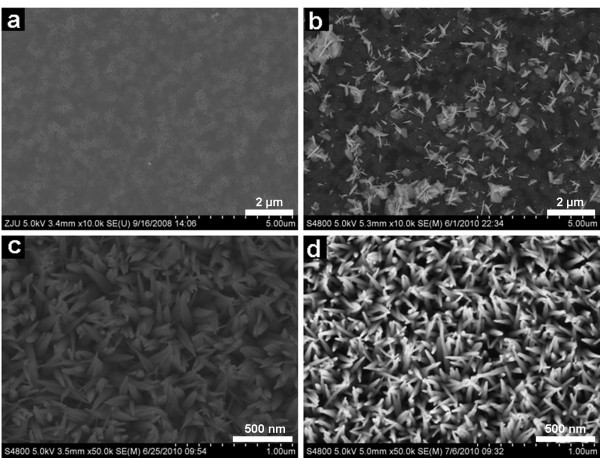
**SEM images of ZnO nanostructures influenced by different Zn(NO_3_)_2 _concentration**: **(a) **0.025 M; **(b) **0.05 M; **(c) **0.1 M; **(d) **0.25 M.

Influences of Zn(NO_3_)_2 _concentrations on the final morphology of nanostructures when the value of *R *was fixed to 1:8 were also investigated, as shown in Figure 2. When the Zn(NO_3_)_2 _concentration was lower than 0.05 M (samples E_1_, E_2_), few or nearly no nanorods formed (Figure 2a,b). Figure 2c shows that the wide and aggregated nanorods appeared if Zn(NO_3_)_2 _concentration was 0.1 M (sample E_3_). The well-aligned nanorod array with sharp tips could only be acquired when the Zn(NO_3_)_2 _concentration reached 0.25 M (Figure 2d).

In a strong alkaline aqueous medium, Zn^2+ ^ions are known to react readily with OH^- ^ions to form Zn(OH)_4_^2- ^complex species which act as the growth units for growing ZnO nanostructures along *c*-axis. Therefore, the main chemical reactions in alkaline zincate solution can simply be represented by the following equations:(1)(2)

Since the existence of ZnO seed-layer can reduce the nucleation energy barrier and the lattice mismatch effectively, pre-coating the substrate with seeds of ZnO provides proper conditions for heterogeneous nucleation and crystal growth.

The final morphologies of the nanostructures are determined by several factors during the growth process. The two most important factors are the concentration of Zn^2+ ^ions and OH^- ^ions. The supersaturation of nonequilibrium growth medium is the key driving force for ZnO nanorods formation. On the one hand, supersaturated OH^- ^in the medium induces sufficient Zn(OH)_4_^2- ^for ZnO crystal growth. Higher Zn(OH)_4_^2- ^concentration will accelerate the reactions to form ZnO. On the other hand, the growth rate along *c*-axis direction can be reduced because superfluous OH^- ^ions are easily adsorbed on the positively charged (0001)-Zn polar surface [32] and eventually lead to the formation of nanosheets (Figure 1a,b). However, for a fix Zn(NO_3_)_2 _concentration, the amount of OH^- ^ions can only be tuned over a small range for producing well-aligned nanorod arrays, in which the shielding effect of OH^- ^ions seems to be weaken compared with the acceleration of the growth rate along the *c*-axis direction. When *R *is too high, zinc hydroxide species form precipitation from the solution; if *R *is too low, the seed-layer would be etched away. Moreover, certain Zn(NO_3_)_2 _concentration is the guarantee of a supersaturated medium for the crystallization because ZnO nanorods cannot grow well with the low concentration of Zn(OH)_4_^2- ^[33].

Under the best condition for the growth of ZnO nanorod, density, and length, controlled growth of ZnO nanorods was investigated. The commonest ways to change the density or the length of nanorods, i.e., the concentrations of Zn(NO_3_)_2 _or OH^- ^ions, are rather hard to be utilized as discussed above. Since it is generally believed that high supersaturation level is favorable for nucleation, whereas low supersaturation level favors crystal growth [34], a supersaturation controlled method is developed. The degree of supersaturation (*S*) is defined as the ratio of the practical concentration (*C*) and the saturated concentration (*C*_s_) of Zn^2+ ^ions in the solution:(3)

The original crystallization with a high ions concentration is generally a nucleation controlled process. For the following process of ZnO nanorods growth, the concentration of Zn^2+ ^ions becomes a controlling factor and the growth mediums with different *S *could influence the dimension and density of arrays.

**Figure 3:**
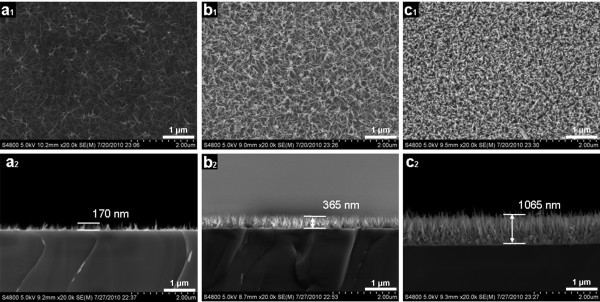
**SEM images of ZnO nanorod arrays with varying the volume of growth media**: **(a_1_) **2 mL (sample A); **(b_1_) **10 mL (sample B); **(c_1_) **20 mL (sample C); cross-sectional SEM images of **(a_2_) **sample A; **(b_2_) **sample B; **(c_2_) **sample C.

With the same ZnO seed-layer and equivalent concentration, the different volume of growth solution strongly influenced the density and length of the ZnO nanorods, as shown in Figure 3. When the ZnO seed-layer was dipped in the 2-mL growth medium and soaked for 2 h at room temperature (approx. 25°C), a relatively sparse, low dimension, and poorly alignment ZnO nanorods were synthesized, with an average length of 170 nm (Figure 3a). In the case of 10-mL growth medium, the higher density of ZnO nanorods compared with sample A is observed in Figure 3b1, with an average length of 365 nm. When the volume of growth solution increased to 20 mL, ZnO nanorods synthesized on substrate were densely packed and well aligned (Figure 3c1). The SEM cross-sectional image (Figure 3c2) shows the ZnO nanorods are 1100 nm in length. These results mean although the initial Zn^2+ ^ions concentrations are exactly the same, while the different absolute quantities of Zn^2+ ^ions (different supersaturation degree) do affect the growth of ZnO nanorods.

In normal conditions, the growth rate slows with time as the Zn^2+ ^ions concentration decrease and eventually equilibrium state in the solution is reached. However, the whole growth processes of ZnO nanorod arrays were in a nonequilibrium state because several hours after taking out samples, there was still precipitation formed from the solution. From the results discussed above, we know that the concentration of Zn(NO_3_)_2 _and the supersaturation degree of solution should be maintained at a certain value to guarantee the nucleation and the growth processes of ZnO. When the initial concentration is same, the nucleation rate should be equivalent in different volume media with the homalographic ZnO-coated substrates. However, the Zn^2+ ^consumption of nucleation process strongly influences the absolute value of Zn^2+ ^ions especially in the minimum growth volume which results in the changing of Zn^2+ ^ions concentration. As the reaction processes, Zn^2+ ^concentration and *S *decrease gradually, but the decreasing rates in different volume media are not the same. AAS results confirm that the final Zn^2+ ^concentrations in 2, 10, and 20 mL medium are 0.087, 0.115, and 0.124 M, respectively. The nucleation and growth rate along the *c*-axis direction are limited by the concentration of the zinc ions in the medium. Therefore, in the growth medium with the smallest volume (2 mL), the nucleation and the growth of ZnO nanorods will quickly stop when the Zn^2+ ^concentration decreases to the certain value. While in the growth medium with larger volume, the nucleation of ZnO can be increased and the nanorod growth can be accelerated.

### Phase and structure of ZnO nanorods

**Figure 4:**
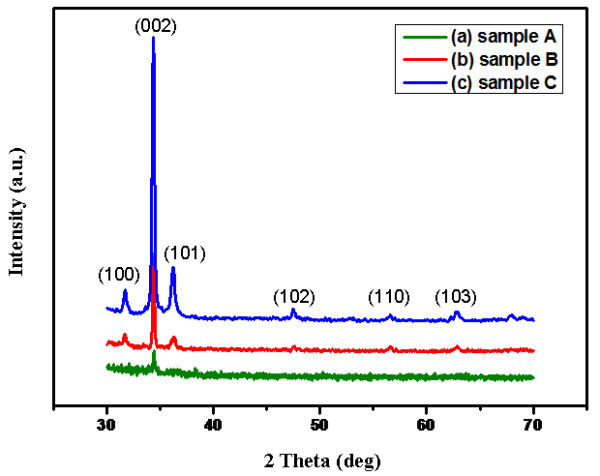
**XRD patterns of ZnO nanorod arrays with different volumes of the growth media**: (a) 2 mL (sample A); (b) 10 mL (sample B); (c) 20 mL (sample C).

The crystallinity of ZnO nanorod arrays grown on Si substrate with different density and length were studied by X-ray diffraction (XRD), as shown in Figure 4. The diffraction peaks in the typical XRD patterns confirm that the ZnO crystals are hexagonal wurtzite structure (*p*6_3_*mc*). A sharp (002) diffraction peak indicates that most of the 1D ZnO nanorods grow preferentially along the [0001] direction (*c*-axis) and perpendicularly to the substrate. In addition, it can be seen that, with the evident enhancement of the peak intensity, the crystalline is abruptly improved, especially indicated by the sharp (002) diffraction peak.

**Figure 5:**
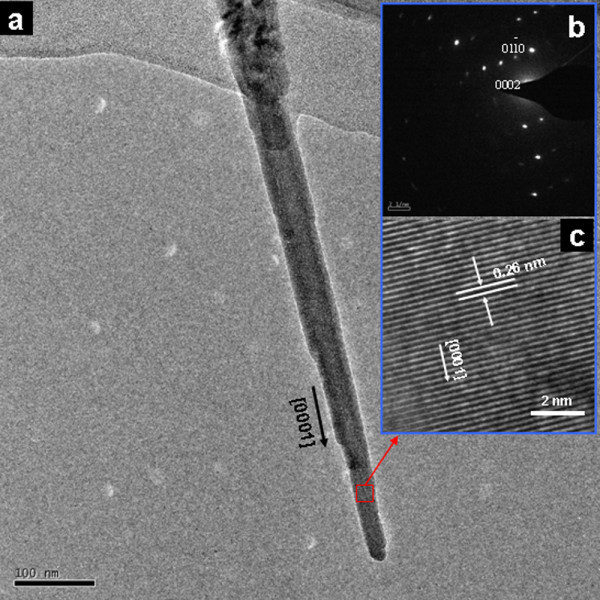
**TEM characterizations of sample C**: (a) TEM image; (b) SAED pattern; (c) HRTEM image.

Figure 5a shows the TEM images of sample C and it can be seen that the diameter difference of nanorod between tip and root is nearly 60 nm. In addition, the selected area electron diffraction (SAED) pattern (Figure 5b) reveals that ZnO nanorods grown at room temperature are monocrystalline. The corresponding region of high-resolution TEM (HRTEM) image (Figure 5c) exhibits the lattice spacing between the adjacent planes is 0.26 nm, which matches well with the (002) crystal planes of wurtzite ZnO. The HRTEM results further confirm that the *c-*axis orientation is the preferential growth direction of the single-crystalline ZnO nanorods which correspond to the XRD results.

### Wettability and electrowetting properties of ZnO nanorod arrays

**Figure 6:**
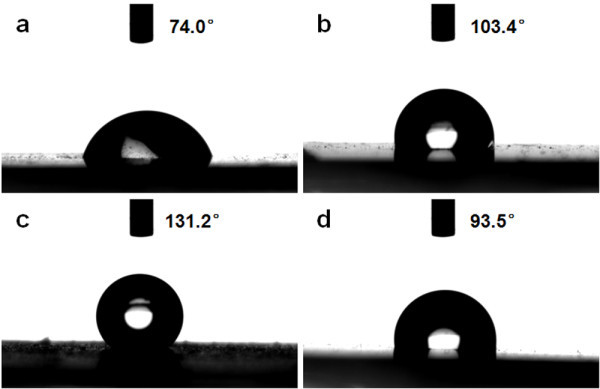
**Photographs of water droplet shape on the surface of (a) sample A; (b) sample B; (c) sample C; and (d) ZnO seed-layer film**.

The superhydrophobic materials have rosen worldwide research interest because of their considerable promise for potential applications, such as self-cleaning surfaces and lab-on-chip devices [35]. It is still a challenge to change the wettability behavior on superhydrophobic surfaces. In this study, we present a comparison of the wettability and electrowetting performances among ZnO nanorod arrays with different densities and dimensions. The wettability measurements of ZnO nanorod arrays on Si substrate with different densities and dimensions (samples A-C) and ZnO seed-layer film show that the water contact angle (*θ*) is 74.0°, 103.4°, 131.2°, and 93.5°, respectively (Figure 6a,b,c,d).

Surface wettability is believed to be regulated by both the chemical composition and the surface geometric structure. Concretely, following the rule of Zisman, wetting surfaces possess high surface energy and nonwetting surfaces are characterized by low surface energy [36]. Furthermore, the Wenzel model predicts that the contact angle of a hydrophilic surface (*θ *< 90°) decreases when its surface is roughened, while roughening a nonwetting surface (*θ *> 90°) always increases its hydrophobicity [37]. In this study, the surface energy could be decreased because the ZnO nanorod arrays, which grow preferentially along *c*-axis direction on the ZnO seed-layer film, and have the lowest surface free energy compared with other random orientations of ZnO films. This can promote the hydrophobicity of surfaces from the rule of Zisman. In addition, the contact angle of ZnO seed-layer film is larger than 90° which could be considered to be hydrophobic. Therefore, the hydrophobic property could be increased when the surface is roughened according to Wenzel model.

In cases of samples B and C, both of the low surface energy and the high roughness enhance the hydrophobicity of surface. With the increase of density and length of ZnO nanorod arrays, the roughness of the nonwetting film surface increases evidently which results in the enhancement of hydrophobicity in samples B and C. However, the wettability of sample A is not consistent with this regulation. It is supposed that the lower crystalline degree and higher oxygen deficiency of short and sparse nanorods increase the surface energy of sample A, which exceeds the effect from surface roughness. Consequently, sample A turn to a hydrophilic surface.

**Figure 7:**
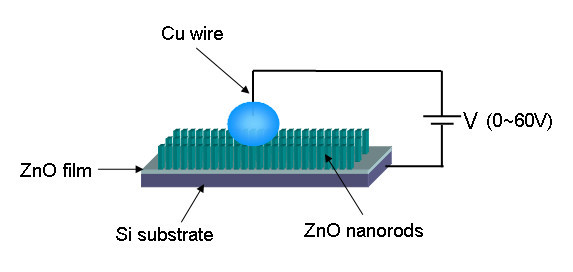
**Sketch of an electrowetting configuration**.

**Figure 8:**
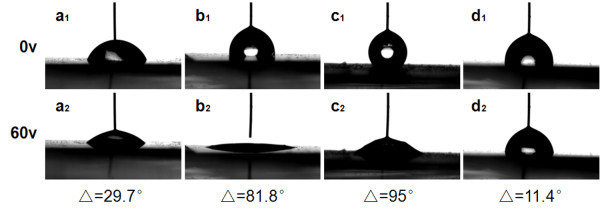
**Photographs of water droplet shape when voltage at 0 V**: **(a_1_) **sample A; **(b_1_) **sample B; **(c_1_) **sample C; and **(d_1_) **ZnO seed-layer film; electrically induced transitions of water droplet shape when voltage at 60 V: **(a_2_) **sample A; **(b_2_) **sample B; **(c_2_) **sample C; and **(d_2_) **ZnO seed-layer film.

**Figure 9:**
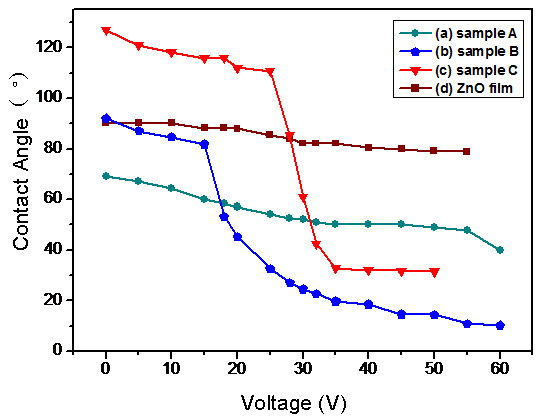
**A gradual electrowetting response curves of contact angle versus applied voltage of (a) sample A; (b) sample B; (c) sample C; (d) ZnO seed-layer film**.

The electrowetting relies on the modification of contact angle by the application of an electrical potential between the conducting substrate and the liquid droplet (Figure 7), which starts from the electrocapillarity phenomenon. For 3-μL droplets of deionized water, the contact angle changes of samples A, B, C, and ZnO seed-layer film were observed by adjusting voltages from 0 to 60 V. Figure 8a1-d1 and 8a2-d2 shows the performance of droplets when voltages at 0 and 60 V were added. As can be observed in Figure 8, the contact angle change of sample C after applying a voltage in the 0 to 60 V range is as large as 95° and the sample A with lower density and length exhibits a poor electrowetting property which only a 29.7° change can be seen. The electric field results in a distribution of charge that alters the surface free energy, causing the droplet to spread on the surface [38]. The lower density of ZnO nanorod array, the fewer free energy changes when apply a voltage, so that the electrowetting property of ZnO film seems to be limited (Figure 8d1,d2). The gradual electrowetting responses were recorded and shown in Figure 9. This gradual response has its advantage for applications such as electrooptics and so on [39].

### Field emission properties of ZnO nanorod arrays

**Figure 10:**
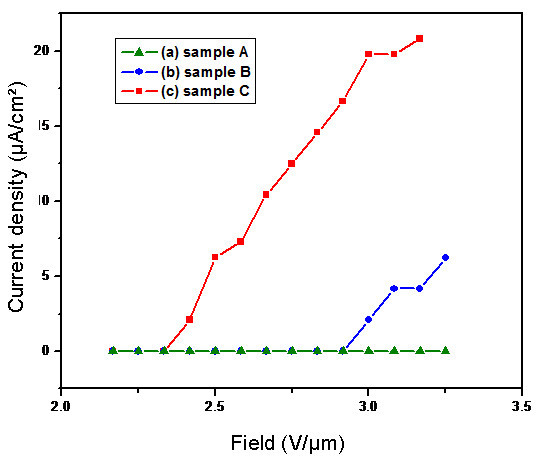
**Field emission properties of (a) sample A; (b) sample B; and (c) sample C**.

It is well known that ZnO 1D nanostructures with different shapes, such as nanowires, nanorods, and nanotubes, have been observed to have better field emission properties than the traditional materials. Commonly, the excellent field emission performance of 1D ZnO nanorod array is believed to benefit from the geometrical configuration of sharp tips, optimized density, and large aspect ratio [4,30]. In this study, we investigated the field emission properties of ZnO nanorod arrays with sharp tips and with different density and dimension prepared on Si substrates. The measurements of field emission were carried out in a vacuum chamber at room temperature. The *J-E *characteristic curves of samples A-C are shown in Figure 10, which gives the field emission current density (*J*) of each sample on the average electric field (*E*) between the anode and the cathode. The results show that samples B and C have obvious and stable field emission properties, and no emission current was detected from sample A even the applied voltage reached nearly 2000 V. The turn-on voltage of samples B and C is about 3.0 and 2.4 V/μm corresponding to a current density of 1 μA/cm^-2^. It is obvious that sample C has the best field emission property with the lowest turn-on electric field and with the highest current density at the same applied electric field on samples. The better field emission performance sample C can be attributed to its high aspect ratio, which was acquired by increasing the medium volume in the present room temperature ZnO nanorods synthesis method.

## Conclusions

In summary, density and dimension controlled ZnO nanorod arrays with sharp tips were synthesized on Si substrates through a simple room temperature solution growth method (approx. 25°C) by pre-forming a ZnO seed-layer on the substrate. Nonequilibrium growth of ZnO nanorod arrays was realized through supersaturation control in room temperature media. Concentrations of growth media, molar ratio of Zn(NO_3_)_2 _to NaOH, and the supersaturation degree are the main factors in density- and length-controlled growth of ZnO nanorods. The water contact angle measurements showed that the density and dimension of nanorods would influence the surface wettability evidently. When applied a voltage in the 0-60 V range on the droplet, the nanorod arrays performed excellent gradual electrowetting responses. The ZnO nanorod array with highest density and dimension revealed the best electrowetting property with the contact angle change of 95°. The field emission property was also optimized by changing the nanorods density and dimension. These demonstrate the present room temperature solution method is an effective way to obtain high-quality controllable ZnO nanorod arrays with potential applications for future nanodevices.

## Abbreviations

AAS: atomic absorption spectroscopy; HRTEM: high-resolution TEM; SAED: selected area electron diffraction; TEM: transmission electron microscope; XRD: X-ray diffraction.

## Competing interests

The authors declare that they have no competing interests.

## Authors' contributions

QL designed and carried out the experiment, statistical analysis and participated in the draft of the manuscript. KC and WW supervised the research and revised the manuscript. CS, PD, GS and GH offered the technique supports. All authors read and approved the final manuscript.

## References

[B1] Ahn CH Mohanta SK Lee NE Cho HK Enhanced exciton-phonon interactions in photoluminescence of ZnO nanopencils Appl Phys Lett 2009 94 261904 10.1063/1.3159829

[B2] Liang WJ Yuhas BD Yang PD Magnetotransport in Co-doped ZnO nanowires Nano Lett 2009 9 892 10.1021/nl8038184 19170557

[B3] Joshi A Aswal DK Gupta SK Yakhmi JV Gangal SA ZnO-nanowires modified polypyrrole films as highly selective and sensitive chlorine sensors Appl Phys Lett 2009 94 103115 10.1063/1.3093499

[B4] Xiao J Zhang XX Zhang GM Field emission from zinc oxide nanotowers: the role of the top morphology Nanotechnology 2008 19 295706 10.1088/0957-4484/19/29/295706 21730611

[B5] Bai XD Wang EG Gao PX Wang ZL Measuring the work function at a nanobelt tip and at a nanoparticle surface Nano Lett 2003 3 1147 10.1021/nl034342p

[B6] Yan CL Liu J Liu F Wu JS Gao K Xue DF Tube formation in nanoscale materials Nanoscale Res Lett 2008 3 473 10.1007/s11671-008-9193-6 20592945 PMC2893443

[B7] Wang XD Song JH Summers CJ Ryou JH Li P Dupuis RD Wang ZL Density-controlled growth of aligned ZnO nanowires sharing a common contact: a simple, low-cost, and mask-free technique for large-scale applications J Phys Chem B 2006 110 7720 10.1021/jp060346h 16610866

[B8] Özgür Ü Alivov Ya I Liu C Teke A Reshchikov MA Doğan S Avrutin V Cho S-J Morkoc H A comprehensive review of ZnO materials and devices J Appl Phys 2005 98 041301 10.1063/1.1992666

[B9] Tian ZR Voigt JA Liu J Mckenzie B Mcdermott MJ Rodriguez MA Konishi H Xu HF Complex and oriented ZnO nanostructures Nat Mater 2003 2 821 10.1038/nmat1014 14634640

[B10] Müller B Riedel M Michel R De Paul SM Hofer R Heger D Grützmacher D Impact of nanometer-scale roughness on contact-angle hysteresis and globulin adsorption J Vac Sci Technol B 2001 19 1715 10.1116/1.1392402

[B11] Lau KKS Bico J Teo KBK Chhowalla M Amaratunga GAJ Milne WI McKinley GH Gleason KK Superhydrophobic carbon nanotube forests Nano Lett 2003 3 1701 10.1021/nl034704t

[B12] Yu HD Zhang ZP Han MY Hao XT Zhu FR A general low-temperature route for large-scale fabrication of highly oriented ZnO nanorod/nanotube arrays J Am Chem Soc 2005 127 2378 10.1021/ja043121y 15724977

[B13] Yang DS Lao CS Zewai AH 4D electron diffraction reveals correlated unidirectional behavior in zinc oxide nanowires Science 2008 321 1660 10.1126/science.1162049 18801993

[B14] Wang XD Summers CJ Wang ZL Large-scale hexagonal-patterned growth of aligned ZnO nanorods for nano-optoelectronics and nanosensor arrays Nano Lett 2004 4 423 10.1021/nl035102c 25427146

[B15] Qurashi A Tabet N Faiz M Yamzaki T Ultra-fast microwave synthesis of ZnO nanowires and their dynamic response toward hydrogen gas Nanoscale Res Lett 2009 4 948 10.1007/s11671-009-9317-7 20596440 PMC2893893

[B16] Galoppini E Rochford JN Chen HH Saraf G Lu YC Hagfeldt A Boschloo G Fast electron transport in metal organic vapor deposition grown dye-sensitized ZnO nanorod solar cells J Phys Chem B 2006 110 16159 10.1021/jp062865q 16913732

[B17] Wang ZL Zinc oxide nanostructures: growth, properties and applications J Phys: Condens Matter 2004 16 829

[B18] Zainelabdin A Zaman S Amin G Nur O Willander M Stable white light electroluminescence from highly flexible polymer/ZnO nanorods hybrid heterojunction grown at 50°C Nanoscale Res Lett 2010 5 1442 10.1007/s11671-010-9659-1 20730076 PMC2920425

[B19] Ahsanulhaq Q Kim JH Kim JH Hahn YB Seedless pattern growth of quasi-aligned ZnO nanorod arrays on cover glass substrates in solution Nanoscale Res Lett 2010 5 669 10.1007/s11671-009-9504-6 PMC2894306 20672029

[B20] Sun HK Luo M Weng WJ Cheng K Du PY Shen G Han GR Position and density control in hydrothermal growth of ZnO nanorod arrays through pre-formed micro/nanodots Nanotechnology 2008 19 395602 10.1088/0957-4484/19/39/395602 21832598

[B21] Jung MH Lee H Selective patterning of ZnO nanorods on silicon substrates using nanoimprint lithography Nanoscale Res Lett 2011 6 159 10.1186/1556-276X-6-159 21711665 PMC3211210

[B22] Look DC Reynolds DC Litton CW Jones RL Eason DB Cantwell G Characterization of homoepitaxial p-type ZnO grown by molecular beam epitaxy Appl Phys Lett 2002 81 1830 10.1063/1.1504875

[B23] Bae J Hong J-I Han WH Choi YJ Snyder RL Superior field emission properties of ZnO nanocones synthesized by pulsed laser deposition Chem Phys Lett 2009 475 260 10.1016/j.cplett.2009.05.045

[B24] Yin ZY Wu SX Zhou XZ Huang X Zhang QC Boey F Zhang H Electrochemical deposition of ZnO nanorods on transparent reduced graphene oxide electrodes for hybrid solar cells Small 2010 6 307 10.1002/smll.200901968 20039255

[B25] Shen GZ Bando Y Liu BD Golberg D Lee C-J Characterization and field-Emission properties of vertically aligned ZnO nanonails and nanopencils fabricated by a modified thermal-evaporation process Adv Funct Mater 2006 16 410 10.1002/adfm.200500571

[B26] Wu XF Lu GW Li C Shi GQ Room-temperature fabrication of highly oriented ZnO nanoneedle arrays by anodization of zinc foil Nanotechnology 2006 17 4936 10.1088/0957-4484/17/19/026

[B27] Cho S Jang J-W Lee J S Lee K-H Room temperature synthesis and optical properties of small diameter (5 nm) ZnO nanorod arrays Nanoscale 2010 2 2199 10.1039/c0nr00278j 20714653

[B28] Qian XM Liu HB Guo YB Song YL Li YL Effect of aspect ratio on field emission properties of ZnO nanorod arrays Nanoscale Res Lett 2008 3 303 10.1007/s11671-008-9154-0 21771350 PMC3244867

[B29] Ma T Guo M Zhang M Zhang YJ Wang XD Density controlled hydrothermal growth of well-aligned ZnO nanorod arrays Nanotechnology 2007 18 035605 10.1088/0957-4484/18/3/035605 19636128

[B30] Pan N Xue HZ Yu MH Cui XF Wang XP Hou JG Huang JX Deng SZ Tip morphology-dependent field emission from ZnO nanorod arrays Nanotechnology 2010 21 225707 10.1088/0957-4484/21/22/225707 20453277

[B31] Zhao Q Zhang HZ Zhu YW Feng SQ Sun XC Xu J Yu DP Morphological effects on the field emission of ZnO nanorod arrays Appl Phys Lett 2005 86 203115 10.1063/1.1931831

[B32] Sun HK Luo M Weng WJ Cheng K Du PY Shen G Han GR Room-temperature preparation of ZnO nanosheets grown on Si substrates by a seed-layer assisted solution route Nanotechnology 2008 19 125603 10.1088/0957-4484/19/12/125603 21817735

[B33] Zhao J Jin ZG Liu XX Liu ZF Growth and morphology of ZnO nanorods prepared from Zn(NO_3_)_2_/NaOH solutions J Europ Ceram Soc 2006 26 3745 10.1016/j.jeurceramsoc.2006.01.006

[B34] Weintraub B Zhou ZZ Li YH Deng YL Solution synthesis of one-dimensional ZnO nanomaterials and their applications Nanoscale 2010 2 1573 10.1039/c0nr00047g 20820688

[B35] Verplanck N Coffinier Y Thomy V Boukherroub R Wettability switching techniques on superhydrophobic surfaces Nanoscale Res Lett 2007 2 577 10.1007/s11671-007-9102-4

[B36] Zisman WA Relation of the equilibrium contact angle to liquid and solid constitution Adv Chem Ser 1964 43 1

[B37] Papadopoulou EL Barberoglou M Zorba V Manousaki A Pagkozidis A Stratakis E Fotakis C Reversible photoinduced wettability transition of hierarchical ZnO structures J Phys Chem C 2009 113 2891 10.1021/jp8085057

[B38] Verheijen HJJ Prins MWJ Reversible electrowetting and trapping of charge: model and experiments Langmuir 1999 15 6616 10.1021/la990548n

[B39] Dhindsa MS Smith NR Heikenfeld J Reversible electrowetting of vertically aligned superhydrophobic carbon nanofibers Langmuir 2006 22 9030 10.1021/la061139b 17014150

